# Mechanics of a Biomimetic Moisture Sensitive Actuator Based on Compression Wood

**DOI:** 10.3390/polym14081624

**Published:** 2022-04-18

**Authors:** Gerhard Sinn, Elisabeth Fizek, Rupert Wimmer, Helga Lichtenegger

**Affiliations:** 1 Department of Material Sciences and Process Engineering, Institute of Physics and Materials Science, University of Natural Resources and Life Sciences, 1190 Vienna, Austria; fizek_elisabeth@hotmail.com (E.F.); helga.lichtenegger@boku.ac.at (H.L.); 2 Department of Material Sciences and Process Engineering, Institute of Wood Technology and Renewable Materials, University of Natural Resources and Life Sciences, 3430 Tulln an der Donau, Austria; rupert.wimmer@boku.ac.at

**Keywords:** actuator, bilayer, moisture, bending, pine, compression wood

## Abstract

Various mechanisms of plant organ movements have been reported, including the close association of two layers with expressed differences in hygroscopic properties. Following this principle, actuator beams composed of thin veneers out of normal and compression wood cut from Scots pine (*Pinus sylvestris* L.) were prepared by using two types of adhesives. The mismatch of the swelling properties of the two layers in tight combination resulted in an expressed bending deflection in response to set humidity changes. The resulting curvatures were measured and analyzed by the Timoshenko bi-metal-model, as well as with an enhanced three-layer model, with the latter also considering the mechanical influence of the glueline on the actuator bending. The thermally induced strain in the original model was replaced by another strain due to moisture changes. The strain was modelled as a function of wood density, along with changes in wood moisture. Experiments with free movement of the bilayer to measure curvature, and with constraints to determine forces, were performed as well. Deformation and magnitude of actuators movements were in close agreement with the enhanced bilayer-model for the phenol-resorcinol-formaldehyde adhesive, which deviated substantially from the casein adhesive glued actuators. The obtained results are seen as critical for wood-based actuator systems that are potentially used in buildings or other applications.

## 1. Introduction

Climate change is and will continue to be one of the greatest challenges for humanity during the decades to come [1]. The reduction of greenhouse gas emissions to mitigate global warming, keeping a temperature rise below 2 °C till the end of this century, is seen as a global goal [2]. In this context energy resilience is important, since 60 to 80% of energy is consumed in urban areas [3]. Energy harvesting technologies are motivated by the desire to address the climate change issue, which concerns the collection of small amounts of ambient energy to power (e.g., wireless devices) [4]. For applications such as shadowing or cooling in the building sectors, low-tech biomimetic principles could be adopted [5], which are also able to harvest the required energy from the environment.

As for actuation, a number of mechanisms can be found in nature, and their competitiveness with technical actuators have been discussed by, for example, Fratzl and Barth [6], or Martone, et al. [7]. Focusing on plants, two principles are most prominent, i.e., osmotic pressurization, hygroscopic swelling and shrinkage [8]. These two principles can be also distinguished with respect to their timescale of movement, since osmotic pressurization takes place faster than hygroscopic swelling. When it comes to rapid movements, mechanical instabilities such as snap-back-buckling or explosive fracture can also be found as actuation principles [9].

Burgert and Fratzl [10] discussed actuation systems in plants with respect to the intrinsic orientation of the cellulose. The orientation of the strong and stiff cellulose fibrils in plant cell walls are strongly influencing the resulting mechanical properties. Water uptake from the environment takes place preferentially between the cellulose microfibrils, inducing swelling and shrinkage perpendicular to the fibril orientation in the first place. Combining tissues having different cellulose orientations permit plants to generate localized strain and therefore movement of such organs [11]. This construction principle can be observed with, for example, pine cones, which open or close under changing humidity conditions [10,12,13]. The construction principle found in pine cones has already been transferred to paper-polymer combinations [14], or into polymer-flax composites with one [15] or two active layers [16]. Natural hygromorphs combining spruce and pine wood have been joined with their longitudinal cell wall axes, oriented perpendicularly to each other (as studied by [17]). Amplitude and response time of the actuators were measured as well as field tests were carried out using larger scale demonstrators. Further upscaling [18,19] trials were performed, demonstrating the practical use, including complex shapes generated for uni- and bi-directional surface curvatures [20].

The design of complex shapes opens new applications in architecture. Bilayers and other actuator principles and their application in architecture have been discussed by López, et al. [21]. The case of hygromorphic materials in architectural design is presented by Holstov, et al. [22], while the climate adaptive building shells were presented by Loonen, et al. [23], including relevant biomimetic principles [5].

So far, not much focus has been put on the glueline, although it is an integral part of the assembly. Vailati, et al. [24] studied the influence of wood adhesives on the kinetics of wooden bilayers. Results on five different adhesives were reported among phenol-resorcinol-formaldehyde (PRF). No long-term influences of the different adhesives were observed, suggesting that the glueline would not have a significant influence on the actuator performance. Nevertheless, the bondline thickness might have an impact on the bending stiffness of the actuator, as well as on the time course of water diffusion through the joint. Glueline thickness and diffusivity are therefore important factors for the design of moisture sensitive actuators.

In this article we have analyzed the behavior of bilayers made of normal and compression wood, coming from the same species with the fibers oriented in parallel. We also have used two types of adhesives. Normal and compression wood differ in their microstructure. The orientation of the microfibrils in the cell walls which is of particular relevance for the swelling properties is discussed in [25,26,27,28,29]. Compression wood is found in the lower part of branches or in tree stems of conifers, which are loaded in compression. Compression wood shows higher microfibril angles in the thickest secondary wall layer S_2_, exhibiting larger swelling rates in the longitudinal direction. It also appears darker due to its higher density, and the cross-sections of the tracheid cells being more rounded instead of hexagonal, with the consequence of higher portions of intercellular spaces [30]. Cell walls of compression wood are thick-walled and highly lignified [31], and often helical fissures are visible [30]. Depending on the grade of compression wood, the S_3_ cell wall layer might be absence [30].

Experiments with moisture induced free movement as well as fixed ends to measure reaction forces were performed. The model presented by Ruggeberg and Burgert [17] for moisture sensitive bilayers was refined by taking into account the glueline and by addressing also wood anatomy and wood density as an integral part of an improved actuator-model.

## 2. Materials and Methods

### 2.1. Actuator Material

A bilayer system from normal and compression wood of Scots pine (*Pinus sylvestris* L.) was used. The normal wood layers were cut from commercially available pine wood, whereas the compression wood samples were prepared from a branch of a standing pine tree. Before sample cutting the materials were stored at standard conditions (65% RH and 20 °C) until weight equilibrium was reached.

Ten equally dimensioned pine wood samples were prepared from identified normal and compression wood. Veneer dimensions were approx. l × w × t = 100 × 20 × 2 mm^3^. The single layers were oriented with the fiber direction L parallel to the sample orientation l, and the radial direction R in direction of the width w.

### 2.2. Sample Gluing

The wooden layers were glued together using two types of adhesives, (1) a bio-based casein glue (CAS), and (2) a high-performance, synthetic, phenol-resorcinol-formaldehyde resin (PRF) adhesive.

The first adhesive type used in this study was a casein glue obtained from Sehestedter Naturfarben^®^ (Sehestedt, Germany), (CAS), which was prepared from milk casein in an aqueous alkaline solvent (7% lime content). The adhesive was prepared according to the manufacturer’s instructions and applied on both surfaces of the adherends.

Casein-glue joints are water resistant but not waterproof [32,33]. The water resistance and strength of casein glue strongly depends on lime (calcium oxide) content [33,34]. A lime content below 10% provides a long pot life and reasonable strong dry bonds on wood but significantly reduces moisture resistance [33]. The shear strength of casein glue, measured by lap shear testing on spruce, was reported to be 7 ± 1.3 MPa from the same product [35].

The second glue was a phenol-resorcinol-formaldehyde resin (PRF, Aerodux 185; Friebe Flugbedarf, curing agent HRP150), with a density of *ρ* = 1.15 ± 0.02 g/cm^3^. PRFs are cold hardening adhesives used mainly for glulam constructions. PRF forms a black glueline, which is water- and weathering resistant and provides high strength [36,37]. The shear strength of PRF measured by Konnerth, Gindl, Harm and Müller [35] on the same product was reported to be 7.8 ± 4.7 MPa, which is slightly stronger than those of CAS.

Wet conditions might soften adhesives and therefore reduce the aforementioned mechanical properties of PRF [38,39] and CAS. For example [40] reported a loss of 50% of casein glue strength after water immersion. The adhesives also differ in their sorption behavior and sorption rates. Wimmer, et al. [41] found that PRF showed lowest diffusion coefficients among all tested wood adhesives (phenol–resorcinol–formaldehyde PRF, melamine-urea-formaldehyde resin MUF, polyvinyl acetate PVA, polyurethane adhesive PUR, and fish gelatin glue).

All samples were glued under wet conditions (85% RH) and fixed between chipboards by using screw clamps for about 24 h and room temperature.

### 2.3. Sorption Cycles

For conditioning a climate exposure test cabinet (WTC Binder, Type 3724009900310; BINDER GmbH, Tuttlingen, Germany) operating at 35 °C was used. The following steps of relative humidity were used: 85, 70, 55, 32, 20 and 0% (oven dried).

#### 2.3.1. Conditioning of Monolayers

On the single veneer plies three de- and adsorption cycles were performed at slow speed to guarantee semi-static conditions. Dimensional changes were measured with a caliper to an accuracy of one hundredth of a millimeter. The masses of the veneers were determined with an analytical scale, after equilibrium moisture contents were reached. From weight change, average sorption isotherms of compression and normal wood were calculated.

#### 2.3.2. Conditioning of Bilayers

Following these semi-static reference measurements, the veneers were paired from the same material at 85% RH one by one with the two kinds of adhesives. This procedure allowed us to predict bilayer properties in a more comprehensive way (since full knowledge of the composing single-layers was available).

After gluing and re-stabilizing the joined samples in the climate chamber the specimens were gripped over a length of 20 mm at one end, with the movement of the free ends being registered by video. Again, three humidity cycles were run. 

Following these experiments, the free ends of two selected actuators were fixed at 85% RH with a load cell (capacity 1000 N), to measure the reaction force as a function of time. Three relative humidity levels were set and one cycle was measured. Force values are important for practical purposes as actuators.

#### 2.3.3. Determining the Radius of Curvature

The radius of curvature *r* of bilayer was determined experimentally from one-side clamped actuators. The free to bend length of the bilayer is l* and the deflection perpendicular to the initially straight actuator at the free end is Δ*h*.

Assuming circular deformation and constant length l* of specimen (neglecting longitudinal swelling) the following relation is obtained:

(1) $$\cos\varphi =\cos\frac{l^{*}}{r}=\frac{r-\Delta h}{r},$$

Using Taylor series expansion of the cosine function the radius of curvature can be approximately calculated as:

(2) $$r\approx \frac{{\left(l^{*}\right)}^{2}}{2\Delta h}$$

### 2.4. Relating Wood Moisture Content and Dimensional Change

As a consequence of moisture uptake or release, wood undergoes dimensional changes. The extent of swelling or shrinkage depends on wood anatomical orientation, and the chemical composition of wood. The wood moisture content *u* is defined as:

(3) $$u=\frac{m_{u}-m_{0}}{m_{0}}$$

where $m_{u}$ is the mass of a piece of wood in equilibrium with its environment at constant humidity conditions, while $m_{0}$ is the oven dry mass.

The wood moisture content *u* is related to the relative humidity (RH) via the sorption isotherm, and in the case of load free movements it is:

(4) u=u(RH)

Within the linear region of the sorption isotherm (approx. 35–85% RH), the function can be linearized by a Taylor series expansion:

(5) $$u\left(RH\right)=u\left(RH_{ref}\right)+\frac{\partial u}{\partial \left(RH\right)}\left(RH-RH_{ref}\right)+𐐃{\left(RH-RH_{ref}\right)}^{2}$$

This linearization was used to fit the sorption isotherm within the humidity range of interest, no distinction was made between ad- and desorption. Sorption isotherms also depend on wood properties such as oven dry density, chemical composition, and anatomy [42].

Moisture adsorption is responsible for swelling, and according to Kollmann and Côté [43] the external volumetric shrinkage might be idealized as a linear function of moisture content *u*, from the oven-dry status, up to *u* = 22 to 24%. At the lower and higher ranges of humidity the volumetric shrinkage is lower and deviates from linearity [42] This relationship holds for different densities: the higher the density, the greater the volumetric shrinkage or swelling. As reported by the same authors [43], maximum volumetric swelling (Equation (6)) is proportional to the product of the moisture content at fibre saturation *u_f_*, and the oven-dry density $\rho_{0}$:

(6) $$\alpha_{Vmax}=\frac{V_{Smax}-V_{D}}{V_{D}}=u_{f}\cdot \rho_{0}$$

with *V_Smax_* being swollen volume at fibre saturation, and *V_D_* the oven dry volume.

Volumetric swelling $\alpha_{V}\left(RH\right)$ at moisture content *u(RH)* can be expressed as fraction of maximum swelling coefficient and related to the maximum linear swelling coefficients, using simple mathematical modifications:

(7) $$\alpha_{v}\left(RH\right)=\alpha_{Vmax}\frac{u\left(RH\right)}{u_{f}}=\frac{V_{S}-V_{D}}{V_{D}}\approx \alpha_{L}+\alpha_{T}+\alpha_{R}$$

Combining Equation (7) with Equation (6), it can be concluded that linear swelling coefficients are also linear functions of wood density. This relation can be expressed as follows:

(8) $$\alpha_{i}\left(RH\right)=a_{imax}\frac{u\left(RH\right)}{u_{f}}=a_{i}\rho_{0\;}u\left(RH\right)$$

$a_{i}$ being correlation coefficients to be determined by the experiments, where the index *i* describes the three anatomical directions, longitudinal L, radial R and tangential T.

For the analysis of the dimensional changes of the veneer strips, knowledge about the wood moisture content is required. As mentioned above, for samples in thermal equilibrium, wood moisture is related to relative humidity via the sorption isotherm (Equation (5)). Combining the aforementioned arguments into a linear regression model, swelling, i.e., moisture induced strain $\varepsilon_{i}\;\left(u\left(RH\right),\rho_{0}\right)$  for the different anatomical directions i∈{L,T,R}, might be modelled as follows:

(9) $$\varepsilon_{i}(u\left(RH\right),\rho_{0})=\frac{l_{i}-l_{0}}{l_{0}}=\alpha_{i}\left(RH\right)=a_{i}\rho_{0}u\left(RH\right)$$

where the right part of Equation (9) corresponds to Equation (8). 

All three anatomical directions *i*, longitudinal L, radial R and tangential T, are analyzed separately.

### 2.5. Modelling the Bilayer Assembly

The analysis of an actuator beam composed of two layers usually follows the argumentation of [44] for a bilayer under thermal expansion. Timoshenko [44] applied the following idealizations for the derivation of his model: (i) the difference in the coefficients of thermal expansion α_i_ remains constant during heating; (ii) friction at the supports can be neglected; and (iii) the width of the strip can be considered as very small. With these assumptions Timoshenko [44] derived an equation for the curvature of a thin bi-metal thermostat with constant actuation strain $\varepsilon_{j}=\alpha_{j}\left(T-T_{0}\right)$ in the single layers *j*. The curvature of the bi-metal strip is then calculated as follows:

(10) $$\begin{array}{ll} \kappa =\frac{1}{r} & =\frac{6{\left(1+m\right)}^{2}}{3{\left(1+m\right)}^{2}+\left(1+mn\right)\left(m^{2}+\frac{1}{mn}\right)}\frac{1}{h}\;\left[\left(\alpha_{2}-\alpha_{1}\right)\left(T-T_{0}\right)\right]\\ & =\frac{f\left(m,n\right)}{h}\Delta \varepsilon \end{array}$$

with

$$m=\frac{h_{2}}{h_{1}},\;h=h_{1}+h_{2},\;n=\frac{E_{2}}{E_{1}}$$

*h_j_* denotes the thickness of the layer indexed *j*; *E_j_* the corresponding Youngs-moduli; thermal expansion coefficients are *α_j_* and *T* and *T*_0_ are actual temperature and original temperature.

The first fraction of Equation (10) depends on the stiffness (geometry as well as Youngs moduli) of the setup, whereas the second factor, the strain difference,

(11) $$\Delta \varepsilon =\left(\alpha_{2}-\alpha_{1}\right)\left(T-T_{0}\right)=\varepsilon_{T2}-\varepsilon_{T1}$$

depends on temperature change and difference in thermal expansion coefficients.

By analogy, for a moisture sensitive actuator the basic relationship from Equation (10) is assumed to remain the same and simply the thermal strains (Equation (11)) have to be replaced by their moisture dependent correspondences. But one has to be aware that the approach to exchange the thermal expansion coefficients with the swelling coefficients and the temperature with the moisture content in the middle part of Equation (11), as it is found, e.g., in Ruggeberg and Burgert [17], is a simplification, since the equivalency is correct only for the difference in the strains, right part of Equation (11). For moisture independent swelling coefficients $\alpha_{1,2}$ resulting error is in the range $\Delta \varepsilon_{err}=-\alpha_{1}\Delta u$, where Δ*u* is the difference in moisture uptake between the two layers. For the bilayer presented in this work the maximum relative error in strain difference reaches 1% of total strain difference. For a perfect, passive and non-hygroscopic layer, the error would be zero, since $\alpha_{1}=0$ and therefore $\Delta \varepsilon_{err}=0$.

Inserting the strains accordingly into Equation (11) yields:

(12) $$\begin{array}{ll} {\Delta \varepsilon }_{M} & =\varepsilon_{2}\left(u\left(RH\right),\rho_{2,0}\right)-\varepsilon_{1}\left(u\left(RH\right),\rho_{1,0}\right)\\ & =a_{2}\cdot \rho_{2,0}\cdot u_{2}\left(RH\right)-a_{1}\cdot \rho_{1,0}\cdot u_{1}\;\left(RH\right) \end{array}$$

In addition, the bilayer approach by Timoshenko [44] has its limitations since it assumes a direct and tied contact between the two layers neglecting any properties of the interface and therefore cannot discriminate between two similar bilayers joined with different glues. Due to this structure the moisture sensitive actuator might be better described including a third layer representing the glue line. Such a three-layer model is e.g., presented by Shapiro and Smela [45], and they got the following formula for the curvature κ:

(13) $$\begin{array}{ll} \kappa =\frac{1}{r} & =\frac{6m_{a}n_{a}\left(1+m_{a}+m_{b}^{2}n_{b}+m_{b}\left(2+m_{a}n_{b}\right)\right)}{\begin{array}{c} 1+m_{b}^{4}n_{b}^{2}+4m_{a}n_{a}+6m_{a}^{2}n_{a}+4m_{a}^{3}n_{a}+m_{a}^{4}n_{a}^{2}\\ +4m_{b}^{3}\left(n_{b}+m_{a}n_{b}n_{a}\right)+6m_{b}^{2}\left(n_{b}+2m_{a}n_{a}+m_{a}^{2}n_{b}n_{a}\right)\\ +4m_{b}\left(n_{b}+3m_{a}\left(1+m_{a}\right)n_{a}+m_{a}^{3}n_{b}n_{a}\right) \end{array}}\cdot \frac{\Delta \varepsilon }{h_{1}}\\ & =f\left(m_{a},n_{a},m_{b},n_{b}\right)\cdot \frac{\Delta \varepsilon }{h_{1}} \end{array}$$

where $m_{a}=\frac{h_{3}}{h_{1}}$, $m_{b}=\frac{h_{2}}{h_{1}}$, $n_{a}=\frac{E_{3}}{E_{1}}$, and $n_{b}=\frac{E_{2}}{E_{1}}$. The layers are numbered from bottom to top with layer 3 being the compression wood layer (a, active), layer 2 the glue line and layer 1 the normal wood layer. The trilayer Equation (13) simplifies to the bilayer-equation Equation (10) when $m_{b}$ is approaching zero.

## 3. Results and Discussion

### 3.1. Wood Density

The average dry density of normal pine wood was determined as $\rho_{n.w.}=\left(588.5\pm 35.0\right)\;\mathrm{kg}/m^{3}$, while that of compression pine wood was $\rho_{c.w.}=\left(746.8\pm 49.4\right)\;\mathrm{kg}/m^{3}$. The density of compression wood is known to be in average 27% higher above the one of normal wood [46]. When the bilayers were assembled the single stripes of normal and compression wood were randomly selected. This resulted in a somewhat uneven wood density distribution between the two sample sets: the density for the PRF bilayers were: $\rho_{n.w.}=\left(579.9\pm 43.7\right)\;\mathrm{kg}/m^{3}$, $\rho_{c.w.}=\left(778.8\pm 18.0\right)\;\mathrm{kg}/m^{3}$ and for the CAS bilayers: $\rho_{n.w.}=\left(597.2\pm 25.7\right)\;\mathrm{kg}/m^{3}$, $\rho_{\mathrm{cw}}=\left(714.7\pm 50.9\right)\;\mathrm{kg}/m^{3}$. These densities were considered in the modeling section.

### 3.2. Sorption Isotherms

**Figure 1:**
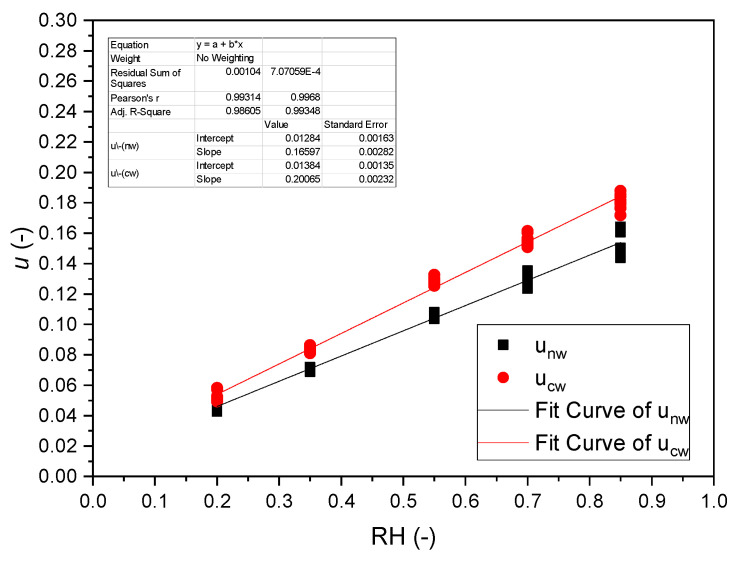
Sorption Isotherms of normal (squares) and compression wood (circles) of reference strips.

Moisture contents were determined according to Equation (3) at 5 relative humidity levels 20, 35, 55, 70, and 85% RH, at a temperature of 35 °C, after equilibrium was reached. Results of the linear regressions are presented in Table 1 as well as in Figure 1. The adjusted coefficients of determination R^2^ > 0.98 are high and justify the linear approximations for the range of relative humidity. The intercept accounts for the nonlinearity of the sorption isotherm at low moisture content, where chemisorption is active [47]. The slope of the sorption isotherm of compression wood is approx. 25% steeper than that of normal wood.

### 3.3. Strains of Reference Material

**Figure 2:**
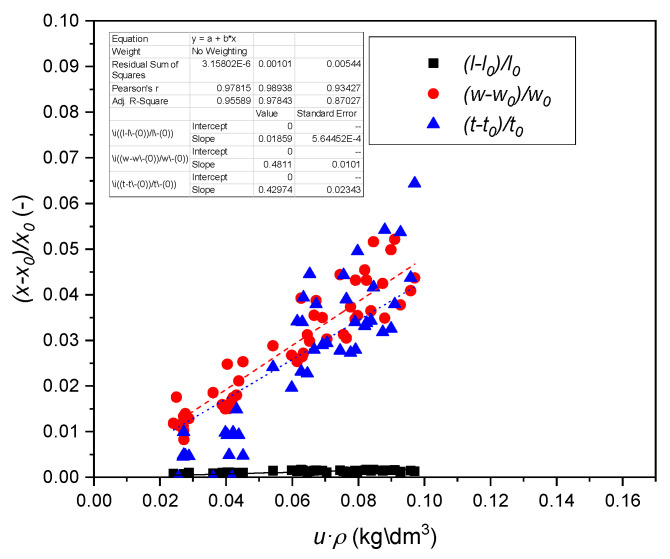
Swelling of normal wood as a function of the product density times moisture content.

**Figure 3:**
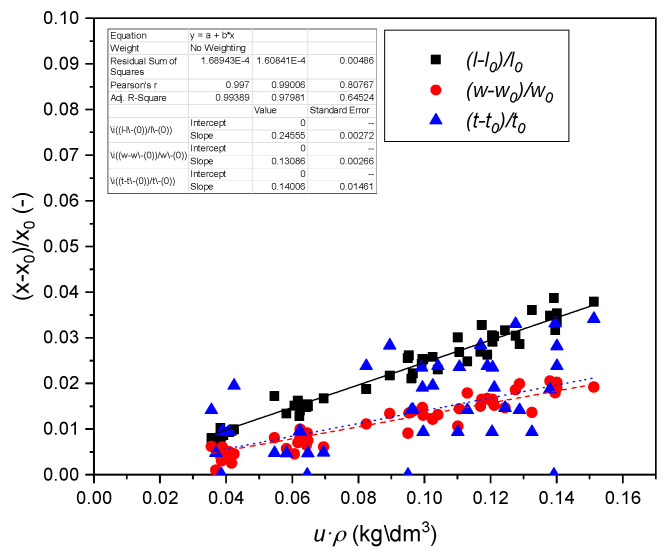
Swelling of compression wood as a function of the product density times moisture content.

The strain $\varepsilon_{L,T,R}$ due to the defined relative humidity change and for a certain oven-dry density was calculated with Equations (8) and (9). The linear regression parameters are summarized in Table 2 and in Figure 2 and Figure 3. Statistically significant correlations were found for all of the slopes correlating the product of moisture content *u* with oven dry density $\rho_{0}$ to the strain for all orientations and wood types. Kollmann and Côté [43] reported that the biggest relative humidity induced movement effects in normal wood are seen in tangential direction, followed by radial and longitudinal direction. In compression wood the biggest dimensional change is seen in the longitudinal direction, followed by the tangential and radial direction, again with minor differences between the radial and tangential direction.

Equation (8) might be used to calculate the approximate maximum swelling coefficients $a_{\mathrm{imax}}$ for $u\left(RH=100\%\right)=u_{f}$.

(14) $$a_{\mathrm{imax}}=a_{i}\cdot \rho_{0}\cdot u_{f}$$

This parameter can be found in literature [17,19,47]. Values from direct regression analysis are summarized in Table 2.

### 3.4. Adhesive Effects

**Figure 4:**
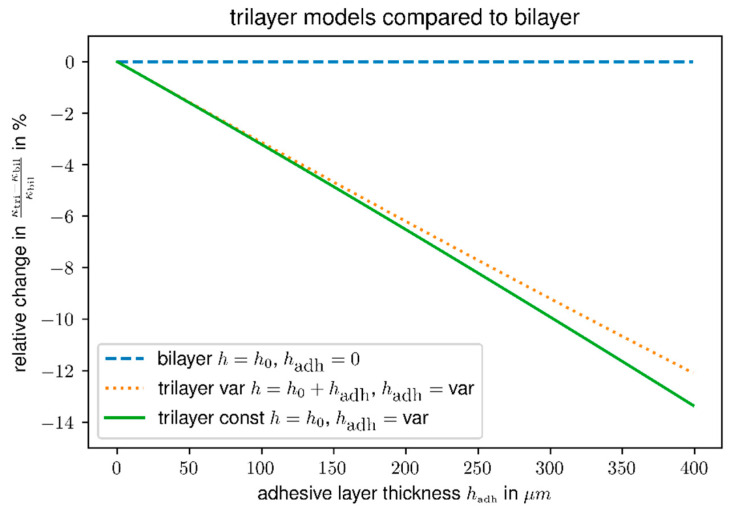
Relative change of curvatures of tri-layer model compared to bilayer model as a function of thickness of adhesive layer: dashed line, bilayer-reference; dotted line, adhesive forms a distinguishable glueline; solid line, adhesive penetrates wood and forms an interlayer.

The average amount of adhesive applied was (0.176 ± 0.092) g for PRF, and (0.299 ± 0.030) g for CAS, respectively. Glueline thicknesses determined by light microscopy were (83.8 ± 29.6) µm for PRF, and (37.6 ± 4.5) µm for CAS. PRF glueline-thicknesses were easy to measure by microscopy, due to their dark appearance and value of 76.5 µm calculated from applied mass was in the range of the thickness measured by microscopy. This was not the case for CAS. The CAS adhesive layers were almost transparent and their glueline thickness measured microscopically deviated significantly from thicknesses estimated from the applied CAS masses. CAS density was assumed to be of similar density as PRF due to lack of experimental data. Estimated glueline thickness of CAS from mas was 142 µm, which was about four times the microscopically measured value. This showed that size and thickness of the glue line could not be exactly determined by the microscopy, especially for the hardly visible casein adhesive. Further, the adhesive penetrated into the wood and formed an interphase of modified properties [48,49]. In case of CAS the interphase might been even thicker than the bondline thickness estimated from adhesive masses, since the volume of the infiltrated interphase is greater than the adhesive volume alone. From the tri-layer-model viewpoint, two extremes can be identified: Either the adhesive forms a distinct glueline and therefore the thickness of the assembly increases proportional to the thickness of the glueline (variable thickness model, more like PRF), or the adhesive infiltrates the specimen completely and the thickness of the assembly remains constant (constant thickness model, more like CAS). As shown in Figure 4 the two systems behave very similar, nevertheless, the constant thickness model, results in a larger loss of curvature than the variable size model. Additionally, it can be concluded from Figure 4, that the absolute thickness of the glue line or interphase has more influence on the curvature than its kind (interphase or distinct line). This is the case for PRF compared to CAS.

### 3.5. Bilayer Curvature

**Figure 5:**
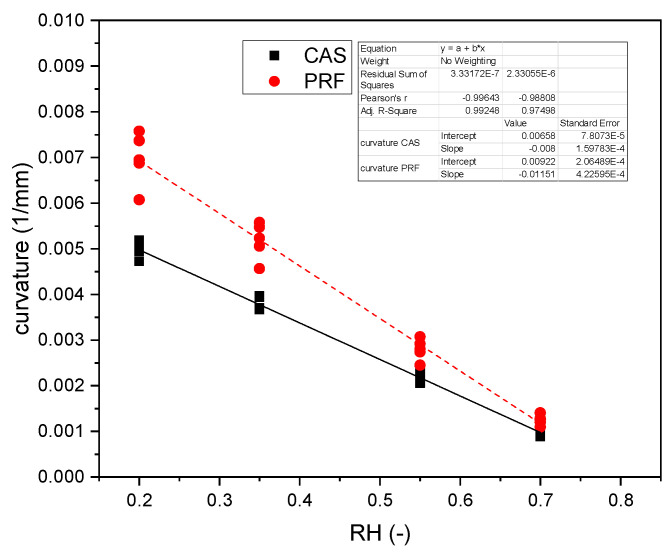
Experimental curvatures for bilayers made with casein adhesive (squares) and PRF adhesive (circles).

To apply Equation (2), a circular deformation was assumed and verified experimentally by photographs. In general, curvature κ was lower for CAS than for the PRF glue. Linear fits show a slope of k_PRF_ = −0.012 RH^−1^mm^−1^ for PRF and k_cas_ = −0.008 RH^−1^mm^−1^ (see Figure 5). The relative difference in slope referenced to CAS is 44%.

### 3.6. Modelling of the Bilayer Assembly

While the experimental set-up showed that the bilayer assembly worked very well as actuator, it is of great interest to predict this behavior for the used raw material.

In a first attempt, the modified Timoshenko-equation (Equation (10)) for a bilayer, where the thermal difference in strain is replaced by a moisture and density dependent strain (Equation (11)) was used to predict the curvature for both systems, the CAS and the PRF-glued bilayers.

**Figure 6:**
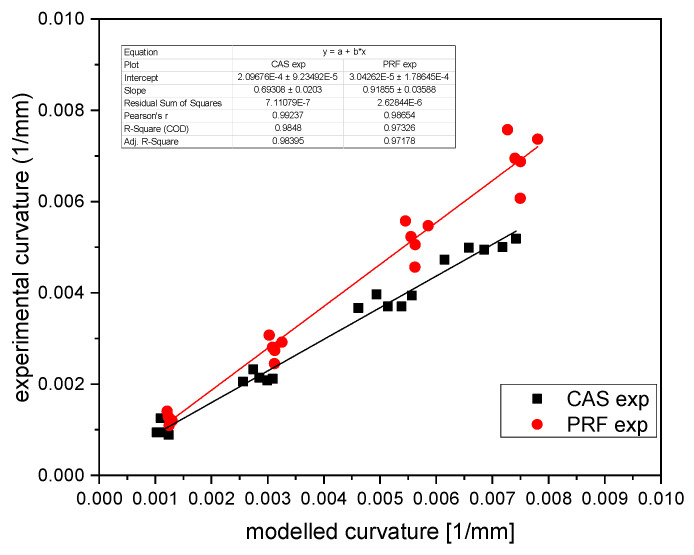
Prediction of simple Timoshenko-model with density-dependent strains.

**Figure 7:**
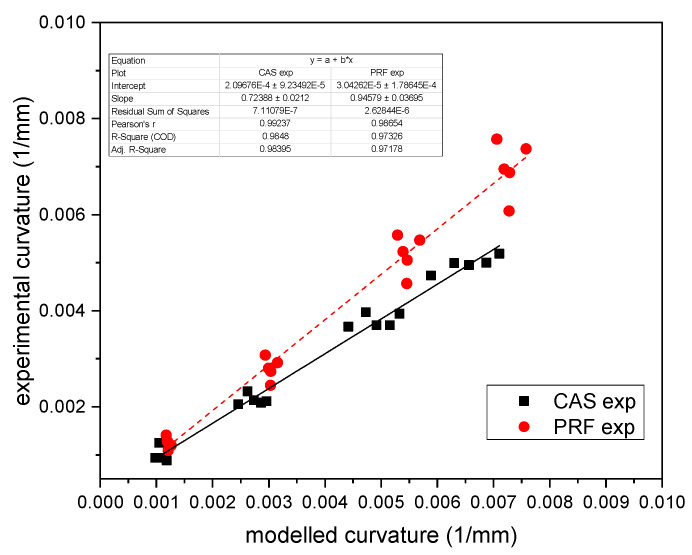
Prediction of multilayer Timoshenko-model with density-dependent strains.

In Figure 6 the experimental results are plotted versus the calculated ones for both systems. The curvature could be predicted with a high coefficient of determination $R^{2}>0.97$ for PRF, the latter having a slightly lower slope of k_PRF_ = 0.919 than the experimental one. The CAS slope reached only in part the expected one (k_cas_ = 0.69), thus showing that the model did not sufficiently represent the experimental differences. Therefore, a three-layer approach was introduced (Equation (13)), with the results shown in Figure 7.

The agreement of theory and experimental data is improved, but the change accounted only for about 4% of the deviation. This can be understood considering that the applied three-layer-model accounts only for a mechanical modification by introducing the glueline as the third layer. The relatively thin middle-layer compared to the thicknesses of the wood veneers and its position close to the neutral axis of deformation can only slightly modify the mechanical response.

Another often non-discussed influence on actuator deformation under moisture change is the change in thickness of the single layers due to swelling or shrinking. A change in thickness modifies the layer-thickness-ratios $m_{i}$ as well as the total thickness of the assembly h. The total thickness appears as $\frac{1}{h}$ in Equations (10) and (13) and therefore a positive relative change in thickness Δh decreases the curvature (i.e., increases the second moment of area or bending stiffness of the beam):

(15) $$\frac{\Delta \kappa }{\kappa }=-\frac{\Delta h}{h}$$

In the evaluation of the model this influence was considered using the moisture sensitive thickness variation from reference samples and by modelling the influence of moisture on moduli of elasticity as straight lines between the wet and the dry states. The following dry values were used $E_{nw}=12\;\mathrm{GPa}$ and $E_{cw}=6\;\mathrm{GPa}$; wet values were measured at 20 °C and 65% rh: $E_{nw\;wet}=6.25\;\mathrm{GPa}$ and $E_{cw\;wet}=3.18\;\mathrm{GPa}$, with nw denoting normal wood and cw compression wood. Using this simplification and separating the models in Equations (10) and (13) for the analysis in two factors: a first factor combining geometry with the moduli of elasticity *f(m,n)/h*, and a second considering the strain difference Δε in longitudinal direction it could be shown that first factor varied only slightly from wet state f(m,n)/h=0.335 to the dry state with f(m,n)/h=0.346 and therefore it could again not also explain the high deviation from the experimental curvature in the case of the CAS group.

Interestingly, these important factors, third adhesive layer and moisture dependent transversal properties of wood were still not sufficient to explain the deviation between the two systems. An explanation might be the different curing behavior of the used adhesives at sample preparation time. Samples were glued under wet conditions (85% RH) and pressed between chipboards by using screw clamps for about 24 h and room temperature. This procedure might have had negative influence on adhesion. CAS is known to soften under high moisture conditions and therefore adhesion might not be succeeded immediately but after some initial drying, allowing the single layers to shrink before fixation and therefore reducing the maximum of moisture induced strain.

### 3.7. Actuator Force

**Figure 8:**
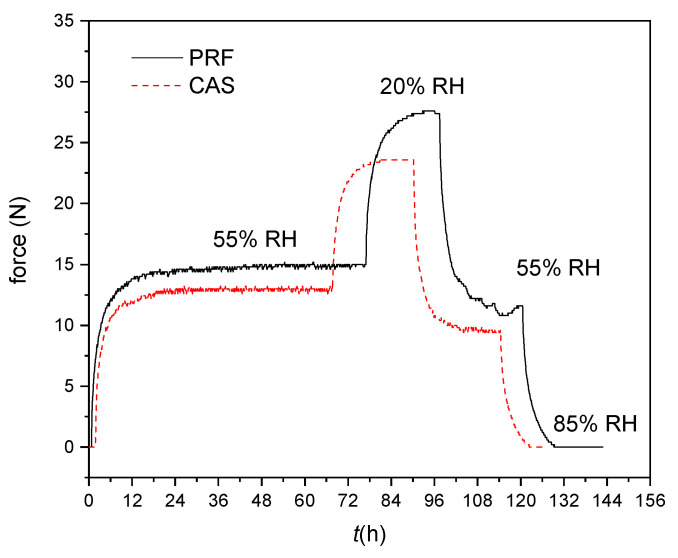
Actuator forces for CAS and PRF bilayers as a function of time and steps of relative humidity.

Two representative force curves for CAS and PRF bilayers as a function of time are shown in Figure 8. The force development over time, going along with the steps in relative humidity is clearly visible. It takes several hours for the bilayers to reach equilibrium. After re-adsorption from 20 to 55% relative humidity the force does not reach the original value but equilibrates at a lower level. This might be explained by two factors: adsorption, which in general reaches a lower moisture level than desorption at the same RH level and adsorption under stress, which is also known to account for lower moisture levels.

The actuator force is again analyzed with the simple model of Timoshenko [44]:

(16) $$F=\frac{3}{2}\frac{EI}{l^{*}}\frac{1}{r}$$

where *E* is the average modulus of elasticity of the two layers and l* the free, not clamped, part of the actuator, *I* the second moment of area and *r* the radius of curvature at a defined moisture level. If we use the corresponding curvatures measured from the free movements of the bilayers at the same relative humidity, we can estimate the modulus of elasticity from the gained force data. The values from least square fitting were 2.5 GPa for CAS and 2.1 GPa for PRF. These values are smaller than the values determined by mechanical testing of the bilayers in tension at 20 °C and 85% RH, where values of E=(3.05±0.14) GPa for CAS and E=(2.72±0.14) GPa for PRF bilayers were measured. Part of this discrepancy might be attributed to the higher temperature of 35 °C, where the bilayers were cycled. The modulus of elasticity of wood is known to decrease with increasing temperature [47].

The combination of normal wood with compression wood as used in our work has the advantage that both materials can be combined along their fiber direction, which is the stiffest and strongest direction of wood. In addition, the proposed bilayer type could find potential applications by using the otherwise low-grade compression wood, as found in branches.

## 4. Conclusions

In this research, moisture sensitive wooden actuators were produced from normal and compression wood of pine. By changing the relative humidity, deformations of the bilayers were observed and measured. Analysis of the system was carried out using the classical Timoshenko model of thermostats and an enhanced tri-layer model. Our comparisons showed that the deformation could be well described qualitatively by a simple numerical model wherein the thermal strain is replaced by moisture induced strain. To improve the model predictions, the influence of density was considered in the calculation of wood moisture content and strain. In case of the casein adhesive glue the curvature was overestimated. This overestimation could be only partially explained by mechanical modelling of the glue line as a third layer. While it could be shown that the thickness of the glue line or interphase had a strong influence on the stiffness of the actuator, moisture dependent moduli and transversal swelling contributed to a minor extend to the deflection. Remaining difference in behavior between the two adhesives might be influenced by additional unknown factors such as the curing behavior of the glue and related stress relaxation. Increased attention must, therefore be paid to the physical conditions during the manufacture of the glue joints, as this could be a source of uncertainty.

The behavior of the reaction force could be well predicted by the deflection of the freely moveable bilayers and is in a suitable range for practical applications. 

The study presented did not address the long-term stability and durability of the bilayers under humidity cycling, while these properties are critical for practical applications and actuator lifetime. In the case of short-term cycling, diffusion properties through the glueline are also of interest as they influence the response characteristics of the actuators. Both points should be addressed in future studies.

## Figures and Tables

**Table 1:** Average linearized sorption isotherms $u\left(RH\right)=u_{0}+\frac{\partial u}{\partial \left(RH\right)}\Delta RH$ (see Equation (5)) of normal wood and compression wood.

Wood Type	Data Points	$u_{0}\left(-\right)$	$\frac{\partial u}{\partial RH}$	R^2^
Normal	50	0.0128 ± 0.0016	0.166 ± 0.003	0.986
Compression	50	0.0138 ± 0.0014	0.207 ± 0.002	0.993

**Table 2:** Linear regression coefficients of normal (n.w.), and compression wood (c.w.) from Equation (6) and linear swelling coefficient α.

Wood	Orientation	Datapoints	*a_i_*, (dm³/kg)	R^2^	$a_{imax}\left(\Delta u\right),$ (% %^−1^)
n.w.	L(l)	60	0.0186 ± 0.0006	0.96	0.011 ± 0.000
	R(w)	60	0.4811 ± 0.0101	0.98	0.282 ± 0.006
	T(t)	60	0.4297 ± 0.0234	0.83	0.252 ± 0.013
c.w.	L(l)	60	0.2456 ± 0.0027	0.99	0.183 ± 0.003
	R(w)	60	0.1309 ± 0.0027	0.98	0.098 ± 0.002
	T(t)	60	0.1401 ± 0.0146	0.32	0.105 ± 0.010

## Data Availability

The data presented in this study are contained within the article.
